# Where Night Torments for Patients Are Absent

**Published:** 1919-01-25

**Authors:** 


### THE EDITOR'S LETTER-BOX.

**[Correspondence on all subjects is invited, but we cannot in any way be responsible for the opinions expressed by our correspondents, who must give their name and address as a guarantee of good faith, but not necessarily for publication. Correspondents are reminded that brevity of style and conciseness of statement greatly facilitate early insertion.]**

### WHERE NIGHT TORMENTS FOR PATIENTS ARE ABSENT.

### A Modern Improved System.

We are indebted to one of the ablest matrons and authorities on nursing affairs for the following interesting and exhaustive account of how one great hospital obviates the employment of first-year probationers on night duty and prevents the tormenting of in-patients in hospital wards during the night. We should welcome further testimony in support of the system so ably dealt with by our correspondent. It would help the world to realise that in the best administered voluntary hospitals the patient is always the first consideration and has at all times the best which devoted women who are nurses can give, and those responsible for their training and duty hours make it their business to secure.—ED. THE HOSPITAL.

*To the Editor of* THE HOSPITAL.

SIR,—Much discussion has taken place of late with regard to nurses' time off duty and the distribution of work during the times on duty. On this point no two hospitals can be alike, with the distinct advantage of maintaining the individuality of the various training-schools which helps to develop the initiative and blending of work in other schools and countries in which those nurses may be called upon to work.

NIGHT DUTY.

At the moment, correspondence has taken place regarding night duty—one fears the story has been written by one who has had an unfortunate and exceptional experience, but one must refuse to believe it is general. Surely the following is the usual custom in most hospitals, based on the consideration that actual ward work is not allocated to night nurses as a rule, and that the comfort of the patients and their welfare are the first considerations. Presuming that night nurses take up their duties at 9.30 p.m., it is usually the custom that without any disturbance from house-men or others for half an hour, the two night nurses (junior and senior) receive full report of the patients from the day sister, the junior night nurse being on call for the needs of any patient.

NIGHT WORK AND SUPERVISION.

At 10 p.m. the senior night nurse makes her first round from bed to bed, to take note of those who may be awake and need attention, adjusting pillows, refilling hot-water bottles, etc.; meanwhile the junior night nurse is setting the kitchen (fire, meals, etc.) for the needs of the night, her only duties (apart from the patients' needs) during the night being presumably her test room, dressing ambulance, test glasses, etc., or such working duties as will not disturb the patients. Meanwhile there may be the visit of a house physician or house surgeon, there may be operations or admissions, should it be the accident or major week of that ward. This with half-hour release for each nurse in turn to take her meal out of the ward, not omitting the early morning tea, coffee, or cocoa before the heavier work is commenced. The four-hourly work—medicines, dressings, etc.—has to be attended to under the supervision and with the assistance of the senior staff nurse. Aperients have to be given with due regard to what is prescribed, at the hours directed, and to the comfort of the patient, with no question of being roused unnecessarily for the same. All this is under the direction of the night superintendent and her assistants, who pay their visits to the wards and who are on call by telephone should emergencies or difficulties arise.

SOME MATERIAL FACTS NOTED.

Now comes the knotty point of breakfast. Before proceeding further attention must be drawn :—

(1) To the fact that no seriously ill patient is disturbed unnecessarily in a well-organised hospital—that if he or she be restless or uncomfortable at an early hour of the morning, it is obvious that a good nurse would wash, dress, and make the bed even at the risk of the process having to be repeated at a later hour of the morning.

(2) The fact must be noted that the ward is closed and lights are out at 8 p.m., and that we are dealing with the workers of the world as hospital patients, whose day begins at an early hour and ends at a late one, if they are the class for which our hospitals exist, hence it is no hardship to have a cup of tea with bread and butter at 5 a.m., nominally called breakfast, no talking being allowed and no one being allowed out of bed till 6 a.m. Washings may be proceeded with at 5.30 quietly and silently, and to the actual comfort of those patients. At 6 a.m. the work of the ward may be commenced, such as bed-making, etc., but in the hospital in which I was trained personally, subject to the ward having only thirty beds, seven beds only were to be made by the night nurses. Again may I draw your attention to the seriously ill cases being completed before this hour for their comfort. Time is of no account, only the patients' comfort.

THE DAY NURSE ARRIVES.

At 7 a.m. the day nurses arrive. Received by the night nurse, who reports any special details, the senior day nurse immediately makes her fifteen beds, with her probationer, but any special or difficult bed may be allocated to a special time or nurse. The senior night nurse proceeds from her seventh bed with her probationer. Meanwhile the third and fourth probationers are allocated to special duties, lunch, etc., and the two junior staff nurses (day and night) complete the washings, dressings, etc.

THE DAY SISTER.

The day sister enters the ward for prayers at 8 a.m., and takes report. The third probationer, with the assistance of a junior staff nurse, proceeds with the "lunches," which are in the nature of breakfast and which in medical wards make for great variety. Night nurses are not responsible for any work in the ward after 8 a.m., and their dinner is served at 9 a.m.

UN-NURSELIKE BEHAVIOUR.

It does not seem just to label the whole nursing profession for un-nurselike behaviour, *i.e.*, noise, talking, visits of house surgeons, disturbing patients for aperients, etc., from isolated examples, perhaps done in ignorance, owing

### THE EDITOR'S LETTER-BOX—(*continued*).

possibly to those in authority not realising the responsibilities of supervision and discipline, the lack of which can only bring unhappiness and discredit on the whole profession.

A GOOD POINT.

Might one here suggest that it would tend to happiness and peaceful working if the terms, discipline, supervision, loyalty, and obedience were more often explained to the nursing staff, not as hardships, but as the bricks which go to build the foundation firmly, on which the true nurse rears her ideals of self-sacrifice and devotion to duty.

TRUE WOMEN AND THE PROFESSION.

With real sympathy for shorter hours, more holidays, and the fuller education which can then demand a higher rate of pay, one is fearful that this trade unionism and the perpetual airing of grievances will drive from the profession the true women who have the welfare of the nursing profession at heart, and who carry with them the silent power which can only command the respect and trust of the most glorious profession a woman can enter.—Yours faithfully,

"MODERN EXPERIENCE."

